# Resting-state functional connectivity indicators of risk and resilience for self-harm in adolescent bipolar disorder

**DOI:** 10.1017/S0033291721005419

**Published:** 2023-06

**Authors:** Mikaela K. Dimick, Megan A. Hird, Alysha A. Sultan, Rachel H. B. Mitchell, Mark Sinyor, Bradley J. MacIntosh, Benjamin I. Goldstein

**Affiliations:** 1 Centre for Youth Bipolar Disorder, Centre for Addiction and Mental Health, Toronto, Ontario, Canada; 2 Department of Pharmacology and Toxicology, University of Toronto Temerty Faculty of Medicine, Toronto, Ontario, Canada; 3 Hurvitz Brain Sciences, Sunnybrook Health Sciences Centre, Toronto, Ontario, Canada; 4 MD Program, University of Toronto Temerty Faculty of Medicine, Toronto, Ontario, Canada; 5 Department of Psychiatry, University of Toronto Temerty Faculty of Medicine, Toronto, Ontario, Canada; 6 Department of Psychiatry, Sunnybrook Health Sciences Centre, Toronto, Ontario, Canada; 7 Department of Medical Biophysics, University of Toronto Temerty Faculty of Medicine, Toronto, Ontario, Canada

**Keywords:** adolescence, bipolar disorder, functional MRI, self-harm

## Abstract

**Background:**

Suicide is the second leading cause of death in all youth and among adults with bipolar disorder (BD). The risk of suicide in BD is among the highest of all psychiatric conditions. Self-harm, including suicide attempts and non-suicidal self-injury, is a leading risk factor for suicide. Neuroimaging studies suggest reward circuits are implicated in both BD and self-harm; however, studies have yet to examine self-harm related resting-state functional connectivity (rsFC) phenotypes within adolescent BD.

**Methods:**

Resting-state fMRI data were analyzed for 141 adolescents, ages 13–20 years, including 38 with BD and lifetime self-harm (BD_SH+_), 33 with BD and no self-harm (BD_SH−_), and 70 healthy controls (HC). The dorsolateral prefrontal cortex (dlPFC), orbitofrontal cortex (OFC) and amygdala were examined as regions of interest in seed-to-voxel analyses. A general linear model was used to explore the bivariate correlations for each seed.

**Results:**

BD_SH−_ had increased positive rsFC between the left amygdala and left lateral occipital cortex, and between the right dlPFC and right frontal pole, and increased negative rsFC between the left amygdala and left superior frontal gyrus compared to BD_SH+_ and HC. BD_SH+_ had increased positive rsFC of the right OFC with the precuneus and left paracingulate gyrus compared to BD_SH−_ and HC.

**Conclusions:**

This study provides preliminary evidence of altered reward-related rsFC in relation to self-harm in adolescents with BD. Between-group differences conveyed a combination of putative risk and resilience connectivity patterns. Future studies are warranted to evaluate changes in rsFC in response to treatment and related changes in self-harm.

## Introduction

Suicide is the second leading cause of death amongst youth ages 10–24 years (Centers for Disease Control and Prevention & National Center for Injury Prevention and Control, 2017). Self-harm, defined as self-damaging acts both with and without suicidal intent, is the strongest predictor of future suicide attempts (Mars et al., 2019; Muehlenkamp, Claes, Havertape, & Plener, 2012). Bipolar disorder (BD), which affects approximately 2% of the population, is a major risk factor for suicide and is present in up to 14% of all suicide deaths, with suicide rates up to 20 times higher than the general population (Latalova, Kamaradova, & Prasko, 2014; Schaffer et al., 2015; Tondo, Isacsson, & Baldessarini, 2003). However, little is known regarding the biological factors underlying the increased risk of suicide in BD (Huber & Yurgelun-Todd, 2019).

Neuroimaging studies examining self-harm across psychiatric disorders implicate reward circuit dysfunction, including the prefrontal cortex, nucleus accumbens, amygdala, and striatum (Haber & Knutson, 2010). Hypersensitivity to reward-relevant stimuli is a key component of the emotion dysregulation that characterizes BD (Henry et al., 2012). Studies of resting-state functional connectivity (rsFC) in youth with BD have implicated anomalous fronto-limbic connectivity (Dickstein et al., 2010; Gao et al., 2014; Kennerley & Walton, 2011; Ridderinkhof, van den Wildenberg, Segalowitz, & Carter, 2004; Singh, Kelley, Chang, & Gotlib, 2015; Stoddard et al., 2015; Tang et al., 2018; Xiao et al., 2013). Task-based functional connectivity studies found youth with a history of self-harm have altered connectivity in reward-related regions including the amygdala, orbitofrontal cortex (OFC), and dorsolateral prefrontal cortex (dlPFC) among others (Auerbach, Pagliaccio, Allison, Alqueza, & Alonso, 2020). Studies examining neurostructure among youth with a history of suicidal ideation and self-harm have found reduced cortical measures in various reward-related regions including the OFC and striatum (Auerbach et al., 2020; Gifuni et al., 2021; Ho et al., 2018, 2021).

Studies examining rsFC among youth with major depressive disorder (MDD) found greater severity of suicidal ideation associated with decreased connectivity between central executive, salience and default mode networks, and decreases in suicidal ideation longitudinally associated with increased connectivity in the salience network (Auerbach et al., 2020). Moreover, among adults with mood disorders, there are differences in rsFC patterns in the default mode, limbic, salience, and central executive networks among those with a history of suicide attempts versus those with only suicidal ideation (Caceda, Bush, James, Stowe, & Kilts, 2018). Studies of adults with BD and MDD have found anomalous functional connectivity in relation to self-harm (Bani-Fatemi et al., 2018; Cheng et al., 2020; Schmaal et al., 2020).

Taken together, reward circuit dysfunction is implicated in both BD and self-harm, self-harm is highly prevalent in BD, and both BD and self-harm each confer an increased risk of suicide in youth, there is a gap of knowledge to date regarding rsFC in relation to self-harm among youth with BD. We therefore examined rsFC in youth with BD, comparing those with a history of self-harm (BD adolescents with a history of self-harm, BD_SH+_) to those without a history of self-harm (BD adolescents without a history of self-harm, BD_SH−_) and HC, in four regions-of-interest (ROIs) within the reward network. We chose to examine the dlPFC, OFC, and amygdala as they have been repeatedly associated with both BD and self-harm (Auerbach et al., 2020; Latalova et al., 2014; Singh et al., 2015). While we hypothesized between-group differences in these prespecified reward-related regions, we did not have *a priori* predictions regarding the direction of these associations. Progress in the understanding of rsFC phenotypes associated with self-harm has the potential to identify treatment targets, and facilitate treatment selection and monitoring, toward the goal of reducing suicidality in BD (Huber & Yurgelun-Todd, 2019).

## Methods

### Participants

Adolescents, ages 13–20, with BD were recruited primarily from a tertiary clinical-research program focused on youth BD. HC adolescents were recruited primarily from the community via advertisements. HC participants had no history of major mood diagnoses, recent anxiety disorders, or any first- or second-degree relatives with BD or psychotic disorder. The presence of any MRI contraindications or recent substance dependence was an exclusion criterion in both groups.

All participants and their parent(s) provided informed consent. This study was approved by the local research ethics board. The authors assert that all procedures contributing to this work comply with the ethical standards of the relevant national and institutional committees on human experimentation and with the Helsinki Declaration of 1975, as revised in 2008. Study participants and their parent(s) were interviewed by a trained interviewer using the Kiddie-Schedule for Affective Disorders–Present and Lifetime version (K-SADS-PL) (Kaufman et al., 1997), a semi-structured diagnostic interview, to collect demographic and clinical information which was performed on the same day as neuroimaging.

History of lifetime self-harm, including a suicide attempt and non-suicidal self-injury (NSSI), was assessed using the Longitudinal Interval Follow-up Evaluation (LIFE) (Keller et al., 1987) Self-Injurious/Suicidal Behavior Scale interview. A suicide attempt was operationally defined as any self-injurious act with a level of the stated intent of at least 3 (‘Definite but still ambivalent’) and a level of medical threat of at least 3 (‘Mild’) on the K-SADS-PL Depression Rating Scale (DRS) (Chambers et al., 1985). Online Supplementary Table S1 includes descriptive anchors for intent and medical threat for the LIFE Self-Injurious/Suicidal Behavior Scale. NSSI was defined as any self-damaging act which did not reach the thresholds for intent and/or medical threat of a suicide attempt. However, if the self-injurious behavior was characteristic of, and better accounted for by, another psychiatric diagnosis (e.g. purging as part of an eating disorder, skin picking, hair pulling), then it was not included. Self-harm was defined as having a history of any self-injurious behavior with or without the intent to end their life. Therefore, participants with a suicide attempt and/or NSSI were categorized as BD_SH+_, and those with no such history were categorized as BD_SH−_.

Participants and their parent(s) were also interviewed for current and most severe lifetime mood episodes using the Mania Rating Scale (MRS) (Axelson et al., 2003) and DRS (Chambers et al., 1985). Diagnoses were based on DSM-IV criteria as this sample was recruited from 2012 through 2017 and the DSM-5 version of K-SADS-PL was not available until December 2016. BD participants met DSM-IV diagnostic criteria for BD-I, BD-II or BD-not otherwise specified (NOS), operationalized as per the Course and Outcome of Bipolar Youth (COBY) study (Axelson et al., 2006). All psychiatric diagnoses were confirmed by a licensed child-adolescent psychiatrist. Anxiety disorders included generalized anxiety disorder, separation anxiety disorder, agoraphobia, and anxiety disorder NOS. Eating disorders included anorexia nervosa, bulimia nervosa, and eating disorder NOS. The Children's Global Assessment Scale (CGAS) was used to obtain overall function in relation to psychiatric symptoms for current (past month), highest past year, and lifetime most severe episode (Shaffer et al., 1983). CGAS scores were rated from 0–100, with higher scores reflecting better functioning. Information regarding lifetime physical and sexual abuse history was obtained from the post-traumatic stress disorder screener within the K-SADS-PL (Kaufman et al., 1997) and from a medical history parent-report containing items querying physical and sexual abuse. Legal history includes any police contact or arrests. The Family History Screen interview was completed for all first- and second-degree relatives to ascertain family psychiatric history (Weissman et al., 2000). Pubertal status was determined using the Pubertal Developmental Scale self-report and reported as Tanner stage (1–5) (Petersen, Crockett, Richards, & Boxer, 1988).

### Magnetic resonance imaging acquisition

Images were acquired on a 3 Tesla Philips Achieva scanner. Structural images were acquired using T1-weighted high-resolution fast-echo imaging (repetition time/echo time/inversion time = 9.5/2.3/1400 ms; spatial resolution 0.94 × 1.17 × 1.2 mm, 256 × 164 × 140 matrix, scan duration 8 m 56 s). Resting-state fMRI was acquired using T2*-weighted echo-planar imaging (TR/TE = 1500/30 ms, flip angle = 70°, ascending slices, a field of view = 230 × 181 mm, spatial resolution = 3 × 3 × 4 mm, matrix 76 × 60 × 28, volumes = 230, scan duration 5 m 50 s). Participants were instructed to rest with their eyes open while staring at a fixation cross and not to focus on any particular thoughts.

### fMRI preprocessing

Preprocessing and analyses were completed using the CONN toolbox (http://www.nitrc.org/projects/conn) (Van Dijk et al., 2010; Whitfield-Gabrieli & Nieto-Castanon, 2012). The first three volumes of functional data were removed in order to account for signal equilibration. The default pipeline for volume-based analyses in the CONN toolbox (Whitfield-Gabrieli & Nieto-Castanon, 2012) was performed for data preprocessing of functional volumes which included the functional realignment and unwarping (participant motion estimation and correction), functional and structural translation, slice-timing correction, functional outlier detection (ART-based identification of outlier scans for scrubbing), functional and structural direct segmentation and normalization to MNI space (simultaneous gray/white/CSF segmentation), and functional smoothing [8 mm FWHM Gaussian filter, using SPM12 (Wellcome Department of Imaging Neuroscience, London, UK; http://www.fil.ion.ucl.ac.uk/spm)]. Head motion was accounted for within the CONN toolbox by identifying problematic timepoints using the Artifact Detection Tools (ART, http://www.nitrc.org/projects/artifact_detect) and via manual inspection of maximum motion at each volume. In ART, we selected the ‘conservative’ setting which defines outlier images as displacement of >0.5 mm from the previous frame in *x*, *y*, or *z* direction, alternatively if the global mean intensity in the image was >3 standard deviation thresholds from mean image intensity for the entire resting scan. In addition, all volumes were manually examined for motion outliers (>2 mm or 2-degree rotation in any direction: *x*, *y*, *z*) and participants were excluded if they had any volumes with motion outliers. A total of 179 adolescents participated in the study, of which 38 were removed due to head motion during the scan (18 BD, 6 BD_SH+_ and 12 BD_SH−_; 20 HC). CONN's default denoising pipeline was used which uses a linear regression of potential confounds including: white matter, CSF, re-alignment, identified outlier scans or scrubbing, and effect of rest (i.e. removing the trend/ramp that is evident at the initiation of the scan session, convolved with hemodynamic response function). Band pass filtering was performed for all functional data (0.008–0.09 Hz). An examination of the histograms from the functional connectivity values for each participant was performed by two independent raters after denoising and revealed normally distributed data for all participants not previously excluded due to motion.

The dlPFC was explored using two seeds, Brodmann area (BA) 9 and BA 46 defined from the BA atlas. The amygdala and OFC seeds were identified using the Harvard-Oxford atlas, generated by the CONN toolbox. The following additional three seed regions defined by the Harvard-Oxford atlas were evaluated in exploratory post-hoc between-group analyses: nucleus accumbens, caudate and putamen. All seeds were parcellated into right and left within the atlases.

### Statistical analysis

Demographics characteristics were compared between the three groups using SPSS Version 26. Group comparisons were made using an analysis of variance (ANOVA) univariate model for continuous variables, and chi-square tests for categorical variables. Clinical characteristics were compared between the BD_SH+_ and BD_SH−_ using *t* tests for continuous variables and chi-squared tests for categorical variables. Statistical significance was set at *p* < 0.05.

**Fig. 1.**
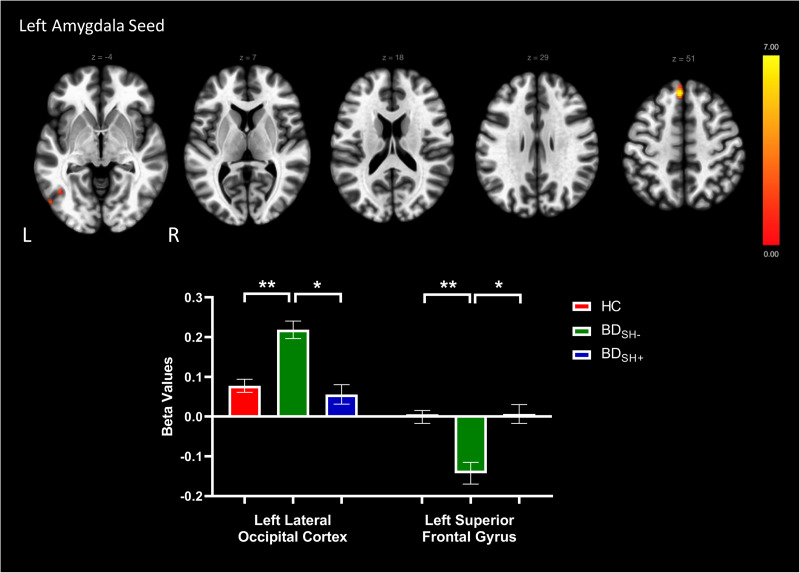
Voxels showing significant connectivity with the left amygdala seed. Graphs showing significant clusters from the left amygdala seed. Beta values correspond to Fischer-transformed correlation coefficient values. Error bars denote the standard error of the mean. *Note*: *p < 0.05, **p < 0.01, ***p < 0.001.

**Fig. 2.**
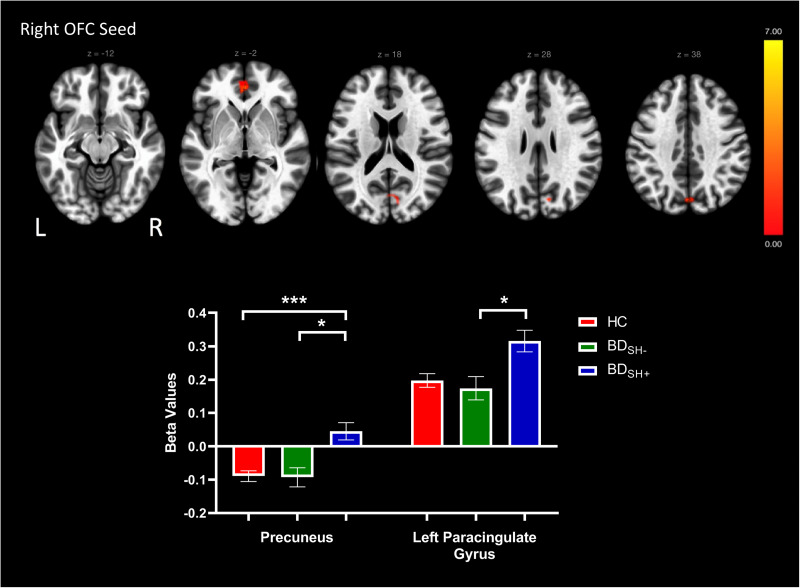
Voxels showing significant connectivity with the right orbitofrontal cortex (OFC) seed. Graphs showing significant clusters from the right OFC seed. Beta values correspond to Fischer-transformed correlation coefficient values. Error bars denote the standard error of the mean. *Note*: *p < 0.05, **p < 0.01, ***p < 0.001.

**Fig. 3.**
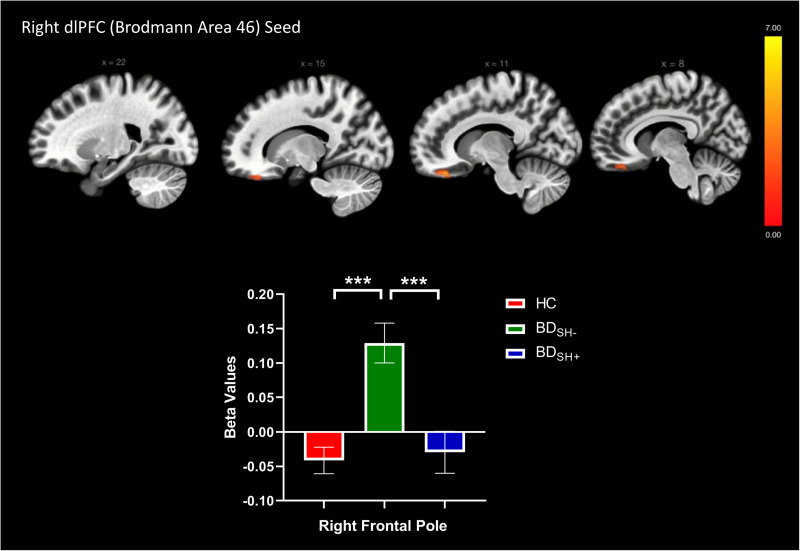
Voxels showing significant connectivity with the right dorsolateral prefrontal cortex (dlPFC) seed (Brodmann Area 46). Graphs showing significant clusters from the right dlPFC seed. Beta values correspond to Fischer-transformed correlation coefficient values. Error bars denote the standard error of the mean. *Note*: *p < 0.05, **p < 0.01, ***p < 0.001.

A seed-to-voxel approach was employed for functional connectivity analyses; Fischer-transformed bivariate correlation coefficients were computed between the timeseries for each bilateral ROI seed and each individual voxel BOLD timeseries to create whole-brain functional connectivity maps. Beta values reported in Figs 1–3 represent Fischer-transformed correlation coefficient values. A general linear model was used to examine the differences between HC versus BD_SH+_ versus BD_SH−_. Second-level analyses of functional connectivity were conducted using multiple regression analyses (voxel-wise *F* statistics) to examine the seed-to-voxel connectivity differences between BD_SH+,_ BD_SH−_ and HC. Age and sex were demeaned and included as covariates in the analyses comparing three groups. Analyses used a voxel size of 3 mm isotropic. Primary analyses used cluster thresholding set at *p* < 0.05 false-discovery rate-corrected, and a more conservative cluster threshold of *p* < 0.01 was used in secondary analyses. Voxel statistical height threshold was set to *p* < 0.001 to identify differences in connectivity between the three groups. For all imaging analyses, Bonferroni correction for multiple comparisons was used to determine significance *p* < 0.01. Significant clusters from the second-level GLM analyses were exported as masks to conduct post-hoc pair-wise comparisons in ROI-to-ROI analyses. Bonferroni correction for multiple comparisons was made (*p* < 0.01) for pairwise post-hoc tests. Four sensitivity analyses were conducted within BD_SH+_ and BD_SH−_ groups to examine the impact of (1) current depression symptoms (2) current mania symptoms (3) current use of lithium [due to putative anti-suicidal properties (Lewitzka et al., 2015)] and (4) current use of second-generation antipsychotics [SGA; due to effects on reward processing via anti-dopaminergic mechanisms (Fervaha et al., 2015)] while controlling for age and sex in ANCOVA models. Medication use was coded in a binary manner (‘0’ for no current use of medication, ‘1’ for current use of medication) and current MRS and DRS scores were mean-centered and used as continuous variables. In addition, a sensitivity analysis was performed examining pubertal status (Tanner stage) between BD_SH+_, BD_SH−_, and HC groups. An ANCOVA model examining rsFC between BD and HC, controlling for age and sex, was also performed for descriptive purposes.

## Results

A total of 141 adolescents were included in analyses: 70 HC, 33 BD_SH−_, and 38 BD_SH+_. The total number of volumes excluded due to motion outliers did not significantly differ by group [mean: BD_SH+_ = 22.52 BD_SH−_ = 25.88, HC = 22.53; F(2, 140) = 0.17, *p* = 0.84]. The average framewise displacement across valid scans also did not differ by group [mean: BD_SH+_ = 0.22 BD_SH−_ = 0.22, HC = 0.20; F(2, 140) = 0.56, *p* = 0.57]. Demographic and clinical characteristics are presented in Table 1. There were more females in the BD_SH+_ compared to BD_SH−_ and HC groups. Tanner stage was higher in BD_SH+_ relative to BD_SH−_ and HC adolescents. BD_SH+_ adolescents had higher BMI and greater proportion of Caucasian race compared to HC. In terms of Children's Global Assessment Score, for current functioning, HC had higher functioning than both BD_SH+_ and BD_SH−_; and for highest functioning score in the past year HC had the highest functioning, followed by BD_SH−_, followed by BD_SH+_. There were no HC participants with a history of self-harm. BD_SH−_ had a higher rate of psychosis and family history of BD compared to BD_SH+_. BD_SH+_ had higher current and the most severe past depression scores, higher current mania rating scores, lower CGAS scores for the highest level of functioning in the past year, and a greater proportion of lifetime suicidal ideation, lifetime eating disorders, and lifetime nicotine use compared to BD_SH−_. In terms of medication use, BD_SH−_ had a higher proportion of participants currently taking lithium compared to BD_SH+_. There were no significant differences between the groups for lifetime medication use.

**Table 1.** Demographic and clinical characteristics

	HC (*n* = 70)	BD_SH−_ (*n* = 33)	BD_SH+_ (*n* = 38)	Statistic (*F*/χ^2^)	*p* Value	Effect size (cramer's *V*/*n*^2^)
Demographics						
Age	17.09 ± 1.70	17.63 ± 1.69	17.29 ± 1.45	1.23	0.3	0.02
SES*	4.40 ± 0.84	4.39 ± 0.79	4.16 ± 0.97	1.94	0.38	0.02
Sex (*n*, % female)	38 (54.3)	14 (42.4)	30 (78.9)	10.54	0.005^a,b^	0.27
Race (*n*, % Caucasian)	39 (55.7)	25 (75.8)	29 (76.3)	6.5	0.04^b^	0.22
Intact family (*n*, %)	43 (61.4)	21 (63.6)	22 (57.9)	0.26	0.89	0.04
BMI (adjusted)	21.93 ± 3.61	23.92 ± 3.61	24.19 ± 5.30	4.74	0.01^b^	0.06
Tanner stage*	4.31 ± 0.60	4.21 ± 0.60	4.63 ± 0.63	11.38	0.003^a,b^	0.05
CGAS: most severe past episode	–	45.41 ± 9.97	42.66 ± 7.80	1.27	0.21	0.15
CGAS: highest past year**	89.16 ± 5.94	72.53 ± 11.62	64.97 ± 10.64	132.80	<0.001^a,c^	0.66
CGAS: past month**	89.09 ± 5.50	67.59 ± 12.02	62.66 ± 10.75	101.36	<0.001^b,c^	0.60
Clinical characteristics, n (%)						
BD-I		14 (42.4)	11 (28.9)	1.7	0.43	0.15
BD-II		10 (30.3)	12 (31.6)			
BD-NOS		9 (27.3)	15 (39.5)			
Age of onset		14.61 ± 3.25	15.02 ± 2.20	0.62	0.54	0.15
Lifetime psychosis		7 (21.2)	2 (5.3)	4.06	0.04	0.24
Lifetime suicide attempts		0 (0)	11 (28.9)			
Lifetime NSSI		0 (0)	35 (92.1)			
Lifetime suicidal ideation		10 (30.3)	32 (84.2)	21.24	<0.001	0.55
Legal history		7 (21.2)	8 (21.1)	0	0.99	0.002
Lifetime physical abuse		1 (3.0)	1 (2.6)	0.01	0.91	0.01
Lifetime sexual abuse		1 (3.0)	2 (5.3)	0.21	0.64	0.06
Lifetime any abuse (physical and/or sexual)		2 (6.1)	2 (5.3)	0.02	0.89	0.01
Lifetime psychiatric hospitalization		15 (45.5)	17 (44.7)	0.004	0.95	0.007
Current depression score		11.55 ± 9.71	19.21 ± 10.69	3.14	0.002	0.75
Lifetime depression score		24.48 ± 12.64	33.92 ± 9.23	3.55	0.001	0.85
Current mania score		4.91 ± 6.82	12.74 ± 10.36	3.81	<0.001	0.89
Lifetime mania score		29.45 ± 10.53	31.00 ± 10.06	0.63	0.53	0.15
Lifetime comorbid diagnoses, n (%)						
ADHD		16 (48.5)	19 (50.0)	0.02	0.90	0.02
Oppositional defiant disorder		6 (18.2)	14 (36.8)	3.04	0.08	0.21
Conduct disorder		1 (3.0)	2 (5.3)	0.22	0.64	0.06
Any anxiety disorder		22 (66.7)	32 (84.2)	2.99	0.08	0.21
Number of anxiety disorders		1.24 ± 1.20	1.74 ± 1.20	1.73	0.09	0.42
Substance use disorder		5 (15.2)	11 (28.9)	1.93	0.17	0.17
Eating disorder		3 (9.1)	17 (44.7)	11.09	0.001	0.40
Nicotine use (yes/no)^a^		1 (3.0)1	10 (26.3)	7.31	0.007	0.32
Family psychiatric history, n (%)						
Mania/hypomania		20 (60.6)	13 (34.2)	4.95	0.03	0.26
Depression		24 (72.7)	28 (73.7)	0.008	0.93	0.01
Anxiety		18 (54.5)	25 (65.8)	0.94	0.33	0.12
ADHD		12 (36.4)	10 (26.3)	0.83	0.36	0.11
Lifetime medications, n (%)						
SGA		26 (78.8)	27 (71.1)	0.56	0.46	0.09
Lithium		10 (30.3)	6 (15.8)	2.13	0.14	0.17
SSRI antidepressants		9 (27.3)	14 (36.8)	0.74	0.39	0.1
Non-SSRI antidepressants		6 (18.2)	7 (18.4)	0.001	0.98	0.003
Stimulants		9 (27.3)	7 (18.4)	0.79	0.37	0.11
Current medications, n (%)						
SGA		21 (63.6)	21 (55.3)	0.51	0.47	0.09
Lithium		9 (27.3)	3 (7.9)	4.72	0.03	0.26
SSRI antidepressants		2 (6.1)	3 (7.9)	0.09	0.76	0.04
Non-SSRI antidepressants		0 (0)	3 (7.9)	2.72	0.10	0.2
Stimulants		3 (9.1)	2 (5.3)	0.4	0.53	0.08

BD, bipolar disorder; CGAS, children's global assessment scale; HC, healthy control; SES, socioeconomic status; BMI, body mass index; s.d., standard deviation; NOS, not otherwise specified; NSSI, non-suicidal self-injury; ADHD, attention deficit-hyperactivity disorder; SGA, second generation antipsychotic; SSRI, selective serotonin reuptake inhibitor. Depression score based on DRS and mania score based on MRS.

*Note*: Values are reported in mean ± standard deviation unless otherwise indicated.

* Kruskal–Wallis test reported.

** Homogeneity of variance violated, Welsh test reported.

Post-hoc pairwise comparisons: a = significant BD_SH+_ *v*. BD_SH−_; b = significant BD_SH+_ *v*. HC; c = significant BD_SH−_ *v*. HC.

### Seed-to-voxel analyses

The HC versus BD_SH+_ versus BD_SH−_ analyses revealed altered rsFC between groups for the left amygdala seed, right OFC seed, and right dlPFC (BA 46) seed (Table 2). Specifically, there was a significant difference in rsFC between the left amygdala seed and a cluster within the left lateral occipital cortex (*p* = 0.002) as well as a cluster within the left superior frontal gyrus (SFG, *p* = 0.002; Fig. 1). Furthermore, between-group differences in rsFC were observed between the right OFC seed and two clusters, including the precuneus (*p* < 0.001) and the left paracingulate gyrus (*p* = 0.04) differed (Fig. 2). Last, there was a significant difference in rsFC between the right dlPFC seed (BA 46) and a cluster within the right frontal pole (*p* = 0.008; Fig. 3). All significant clusters except the left paracingulate gyrus survived cluster thresholding at *p* < 0.01. There were no significant differences in rsFC originating from the right amygdala, left OFC, left dlPFC (BA 46), or bilateral dlPFC (BA 9) seeds.

**Table 2.** Characteristics of significant rsFC clusters

Seeds	MNI coordinates			Cluster size (voxels)	Cluster size (mm^3^)	*p* FDR corrected	Main region	Additional region(s)
	*x*	*y*	*z*					
Left amygdala	−56	−72	−6	170	4590	0.002	Left Lateral Occipital Cortex	Left Inferior Temporal and Middle Temporal Gyri
	−4	36	52	194	5238	0.002	Left Superior Frontal Gyrus	Left Frontal Pole
Right dlPFC, BA 46	12	36	−34	143	3861	0.008	Right Frontal Pole	Frontal Medial and Right Frontal Orbital Cortices
Right OFC	0	−76	36	235	6345	<0.001	Precuneus	Right Cuneal, Right Intracalcarine, Left Cuneal, and Supracalcarine Cortices
	−2	44	−2	100	2700	0.04*	Left Paracingulate Gyrus	Anterior Cingulate and Right Paracingulate Gyri

*Note*: BA, Brodmann Area; MNI, Montreal Neurological Institute; FDR, False Discovery Rate; dlPFC, dorsolateral prefrontal cortex; OFC, orbitofrontal cortex; rsFC, resting-state functional connectivity.

* Did not survive cluster thresholding *p* < 0.01.

Results for the descriptive BD versus HC analysis are presented in online Supplementary Table S2.

### Post-hoc ROI-to-ROI analyses

Significant clusters from seed-to-voxel analyses were exported as masks to conduct ROI-to-ROI post-hoc pairwise comparisons. BD_SH−_ showed significantly higher anti-correlation between the left amygdala and left SFG compared to BD_SH+_ and HC. Furthermore, BD_SH−_ showed significantly increased connectivity between the left amygdala and left lateral occipital cortex compared to BD_SH+_ and HC. There were no significant differences between BD_SH+_ and HC for the amygdala seed ROI-to-ROI analyses.

BD_SH+_ showed significantly higher anti-correlation between the right OFC seed and the precuneus compared to BD_SH−_ and HC. BD_SH+_ showed significantly higher positive connectivity between the right OFC seed and the left paracingulate gyrus compared to BD_SH−_ and HC. There were no significant differences between BD_SH−_ and HC for the right OFC seed ROI-to-ROI analyses.

BD_SH−_ showed significantly higher anti-correlation between the right dlPFC (BA 46) seed and the right frontal pole compared to BD_SH+_ and HC. There were no significant differences between BD_SH+_ and HC in right dlPFC (BA 46) seed ROI-to-ROI analyses.

Clusters identified in the main analysis remained significant when controlling for the role of medications (SGA, lithium) and mood (current DRS score, current MRS score) within BD groups. In sensitivity analyses controlling for pubertal status within all three groups, all clusters from the main analyses remained significant. The average framewise displacement across scans had a significant small correlation with connectivity results between the left amygdala seed and left superior frontal gyrus cluster (*r*^2^ = 0.19, *p* = 0.03). There were no other significant correlations between average framewise displacement and connectivity patterns from the primary results.

### Exploratory post-hoc analyses

There was altered connectivity between the three groups from the left nucleus accumbens seed to the left superior parietal lobule (cluster size: 331; MNI coordinates *x*: −40, *y*: −52, *z*: 56; *p* = 0.00002; online Supplementary Fig. S1) which was significant at cluster thresholding of *p* < 0.01. There were no significant between-group differences in functional connectivity from the right nucleus accumbens, bilateral caudate, and bilateral putamen seeds. In post-hoc pairwise comparisons, HC showed significantly higher anti-correlation between the left nucleus accumbens and the left superior parietal lobule compared to BD_SH+_ and BD_SH−_. There were no significant differences between BD_SH+_ and BD_SH−_.

## Discussion

This study employed a seed-to-voxel approach to investigate patterns of differential reward circuit rsFC among BD_SH+,_ BD_SH−_ and HC adolescents. Results revealed between-group differences in rsFC among three seeds in the reward circuit: the left amygdala, right OFC, and right dlPFC (BA 46). First, we found increased connectivity between the left amygdala seed and the left lateral occipital cortex and decreased connectivity between the left amygdala seed and the left SFG in BD_SH−_ relative to BD_SH+_ and HC adolescents. Second, we observed increased connectivity from the right OFC seed to the precuneus and left paracingulate gyrus in BD_SH+_ compared to BD_SH−_. Third, we found increased connectivity between the right dlPFC (BA 46) and the right frontal pole in BD_SH−_ relative to BD_SH+_ and HC adolescents. This study represents the only study to investigate the rsFC of self-harm within youth BD. Two unique patterns of altered reward circuit connectivity among the BD groups emerged from our findings: connectivity from the OFC seed was different in BD_SH+_ as compared to both BD_SH−_ and HC adolescents, reflecting a putative neurofunctional indicator of risk; and connectivity from the amygdala seed and dlPFC seed (BA 46) was different in BD_SH−_ as compared to both BD_SH+_ and HC adolescents, reflecting a putative neurofunctional indicator of resilience.

The paucity of studies investigating rsFC associated with self-harm in BD provides a limited basis for contextualizing current findings. Two prior studies have examined the rsFC of self-harm using combined samples of adults with MDD and BD, neither of which has examined the reward circuit. One study found that individuals with a history of suicide attempts had higher connectivity between the habenula and right amygdala (in addition to other regions) compared to those without a history of suicide attempts and HCs (Ambrosi et al., 2019). The second study found connectivity patterns between the default mode network and the limbic, salience and central executive networks, differentiated participants with a recent suicide attempt from participants with suicidal ideation but no recent self-harm (Caceda et al., 2018). There has only been one study examining rsFC of self-harm within BD, showing differences in connectivity in the precuneus, insula, and superior temporal gyrus between those with and without a history of suicide attempts (Cheng et al., 2020).

We found increased positive connectivity between the right OFC seed and the precuneus among BD_SH+_, implicated in our findings as a putative risk indicator. The precuneus is involved in a variety of highly complex tasks, including self-referential processing (Cavanna & Trimble, 2006). A prior study showed that youth with MDD and a history of self-harm had greater rsFC between the precuneus and the SFG among other regions (Auerbach et al., 2020). The precuneus has been found to have decreased global brain connectivity in BD-I adults with a history of suicide attempts relative to no suicide attempt (Cheng et al., 2020).

There was increased connectivity from the left amygdala seed to the left lateral occipital cortex and decreased connectivity to the left SFG in BD_SH−_ relative to BD_SH+_ and HC adolescents. A similar pattern emerged between the right dlPFC (BA 46) seed and the right frontal pole. These findings were somewhat unexpected, given that we hypothesized that BD_SH+_ would have altered connectivity relative to the other two groups. While we recognize the limitations of a cross-sectional study, we speculate that this finding might reflect a putative compensatory mechanism of the BD_SH−_ group which may be protective against self-harm. A prior study similarly found depressed youth without a history of suicide attempts had different activation patterns from HC during an Iowa Gambling Task, whereas depressed youth with a prior suicide attempt did not differ from HC (Auerbach et al., 2020). Although significant brain regions in the prior study differed from those identified in the current study findings, there is a similar pattern suggestive of a protective rsFC phenotype for adolescents with a mood disorder without a history of self-harm.

The significant clusters identified in this study are located in brain regions involved in various neurocognitive functions relevant to BD. The SFG, a key region involved in working memory (du Boisgueheneuc et al., 2006), was found to have decreased connectivity to the left amygdala seed among BD_SH−_, potentially representing an adaptive biological marker of resilience. Similar to our findings of negative connectivity among those without a history of self-harm, a prior study found that adult men with MDD and history of a suicide attempt had increased neural activity within the OFC and decreased activity in the SFG during exposure to angry faces compared to those without a prior suicide attempt (Jollant et al., 2008). The right frontal pole was found to have decreased connectivity to the right dlPFC (BA 46) seed among BD_SH−_, a similar pattern to our left amygdala seed findings. A prior study found lower rsFC between the amygdala and right frontal pole among adolescents with NSSI compared to HC (Auerbach et al., 2020). Furthermore, increased frontal pole volume predicted suicide attempts in a sample of females with BD (Bani-Fatemi et al., 2018). Prior studies have contrasted our observed pattern of negative connectivity in adolescents without a history of self-harm. In a study of youth with BD, those *with* a history of suicide attempts had decreased connectivity between the amygdala and OFC during happy and neutral face conditions (Auerbach et al., 2020); the same pattern was observed in adults with MDD and a history of suicide attempts, who showed decreased activation in left OFC and left occipital cortex compared to both patient controls and HC (Jollant et al., 2010).

In exploratory analyses, we examined additional subcortical reward-related regions. We found that BD youth with and without a history of self-harm had decreased functional connectivity from the left nucleus accumbens seed to the left superior parietal lobule compared to HC youth. There was no difference between BD youth with and without a history of self-harm, limiting the interpretation that this finding may be related to self-harm. The nucleus accumbens is part of the ventral striatum, which is a key region in reward circuitry and implicated in self-harm (Schmaal et al., 2020). A study of female youth with a history of NSSI found that reduction of NSSI following treatment with N-acetylcysteine (NAC) was associated with decreased functional connectivity between the nucleus accumbens and the superior medial frontal cortex (Cullen et al., 2020). While the current study did not focus on demographic and clinical differences between the BD_SH+_ and BD_SH−_ groups, some differences that emerged warrant comment. There was a higher proportion of females in the BD_SH+_ group, as could be expected based on the clinical epidemiology of self-harm (Mars et al., 2019). While the BD_SH+_ group was younger, pubertal stages were higher in this group, which may be attributable to earlier puberty in females. Of note, all findings remained significant in sensitivity analyses controlling for the pubertal stage. Interestingly, reward-related clinical characteristics were more common in the BD_SH+_ group, including comorbid eating disorders, higher BMI, and history of nicotine use.

The findings of this study should be interpreted in the context of several limitations. First, the cross-sectional design precludes any inferences of causation or directionality. Longitudinal studies are needed to elucidate whether these connectivity patterns precede self-harm, and whether they vary over time and/or in relation to mood or suicidality. Second, the observational design may not be as sensitive as experimental paradigm approaches probing responses to emotion, reward, and/or suicide-related tasks. Third, we selected regions of interest that were most strongly supported by prior studies in both youth BD and self-harm, and recognize that there are other potential seeds of interest such as the insular cortex, ventrolateral PFC, and habenula. Fourth, this study examined a single analytic approach and a single neuroimaging phenotype. Future studies examining independent component analyses of rsFC, diffusion tensor imaging, gray matter structure, and cerebral blood flow are needed to provide further insights regarding the brain circuits, structures, and processes involved in self-harm. Fifth, as with most BD studies, there was substantial heterogeneity within our BD sample in terms of medication status and clinical profile (i.e. current mood state, BD subtype, psychiatric comorbidity, family psychiatric history). While more homogeneous approaches offer certain advantages, our goal was to generate findings that are broadly relevant in clinical populations, which are uniformly characterized by such heterogeneity. Sixth, despite research linking specific cognitive processes with functional brain regions, our inferences about cognitive processes involved in our findings are tentative and task-based fMRI studies are needed to confirm these associations. Last, this study was not sufficiently powered to examine functional connectivity of suicide attempts and NSSI separately, and there may be important phenotypic differences between these behaviors. In addition to intent, which is part of the distinction among NSSI and suicide attempts, the frequency and medical severity of self-harm warrants evaluation in future studies. Future studies with larger sample sizes are needed to examine these behaviors and related characteristics separately.

## Conclusion

In summary, this study found two consistent patterns of rsFC related to self-harm. The first pattern can be characterized as a putative indicator of self-harm risk: BD_SH+_ had increased connectivity compared to BD_SH−_ and HC adolescents from the OFC to the precuneus and cingulate cortex. The second pattern might be characterized as putatively resilient: BD_SH−_ adolescents demonstrated greater connectivity between the (1) amygdala seed and occipital and frontal regions, and (2) dlPFC seed and frontal regions compared to both BD_SH+_ and HC adolescents. To our knowledge, there have been no prior studies examining the rsFC of self-harm within adolescent BD. As such, this study provides preliminary inferences regarding the neurobiology of self-harm among adolescents with BD, a group at extraordinarily high risk of suicide. With continued efforts, this line of research has the potential to yield objective indicators of self-harm risk that might assist with risk stratification and, ultimately, influence the process of selecting, targeting, and monitoring the effects of various preventive and treatment interventions for self-harm. In the interim, present findings may help reduce the blame, bias, and disadvantage faced by adolescents with mood disorders and self-harm (Cvinar, 2005).

## Supplementary material

For supplementary material accompanying this paper visit https://doi.org/10.1017/S0033291721005419. click here to view supplementary material
