# Supplementary material for: Resting-state functional connectivity indicators of risk and resilience for self-harm in adolescent bipolar disorder
Source: Psychol Med. 2022 Mar 8;53(8):3377–86. doi: 10.1017/S0033291721005419 (PMC10277718; doi:10.1017/S0033291721005419)
Supplement: Supplementary file 1 [file S0033291721005419sup001.docx]

**Supplementary Materials**

**Supplementary Table 1.** Measurement scale for level of intent and medical threat of suicidal behaviors.

| **Intent** | |
| --- | --- |
| 0 | No information |
| 1 | Obviously no intent |
| 2 | Only minimal intent |
| 3 | Definite but still ambivalent |
| 4 | Serious |
| 5 | Very serious |
| 6 | Extreme (e.g., careful planning and every expectation of death) |
| **Medical Threat** | |
| 0 | No information |
| 1 | No danger (e.g. no effect – held pills in hand) |
| 2 | Minimal (e.g. scratch on wrist) |
| 3 | Mild (e.g. took ten aspirins – mild gastritis) |
| 4 | Moderate (e.g. took ten secobarbital sodium – briefly unconscious) |
| 5 | Severe (e.g. cut throat) |
| 6 | Extreme (e.g. respiratory arrest or prolonged coma) |
| 7 | Death |

**Supplementary Table 2.** Characteristics of significant rsFC clusters between BD and HC.

| **Seeds** | **MNI Coordinates** | | | **Cluster**  **Size (Voxels)** | **Cluster**  **Size (mm^3^)** | ***p* FDR corrected** | **Main Region** | **Additional Region(s)** |
| --- | --- | --- | --- | --- | --- | --- | --- | --- |
|  | **x** | **y** | **z** |  |  |  |  |  |
| Left Amygdala | No significant clusters | | | | | | | |
| Right Amygdala | No significant clusters | | | | | | | |
| Left dlPFC, BA 46 | -38 | -54 | -4 | 106 | 2862 | 0.04** | Left Inferior Temporal Gyrus | Left Temporal Occipital Fusiform Cortex |
| Right dlPFC, BA 46 | No significant clusters | | | | | | | |
| Left dlPFC, BA 9 | No significant clusters | | | | | | | |
| Right dlPFC, BA 9 | No significant clusters | | | | | | | |
| Left OFC | No significant clusters | | | | | | | |
| Right OFC | No significant clusters | | | | | | | |

***Note:** BA = Brodmann Area; MNI = Montreal Neurological Institute; FDR = False Discovery Rate; dlPFC = dorsolateral prefrontal cortex; OFC = orbitofrontal cortex; rsFC = resting-state functional connectivity.

**Did not survive cluster thresholding at *p*<0.01.


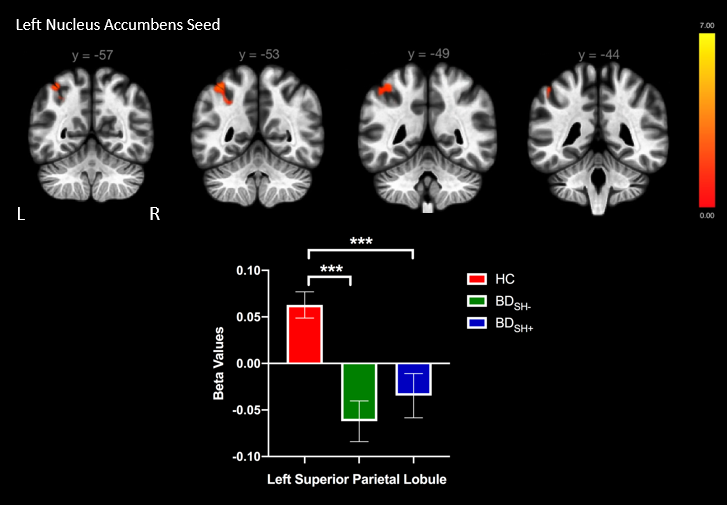


**Supplementary Figure 1.** Voxels showing significant connectivity with the left nucleus accumbens seed. Graphs showing significant clusters from left nucleus accumbens seed. Beta values correspond to Fischer-transformed correlation coefficient values. Error bars denote standard error of the mean.

**Note**: *p<0.05, **p<0.01, ***p<0.001
